# A single-center randomized controlled trial observing the safety and efficacy of modified step-up graded Valsalva manoeuver in patients with vasovagal syncope

**DOI:** 10.1371/journal.pone.0191880

**Published:** 2018-01-30

**Authors:** Li He, Lan Wang, Lun Li, Xiaoyan Liu, Yijun Yu, Xiaoyun Zeng, Huanhuan Li, Ye Gu

**Affiliations:** 1 Department of Cardiology, Puai Hospital, Huazhong University of Science and Technology, Wuhan, Hubei province, China; 2 Department of Neurology, Puai Hospital, Huazhong University of Science and Technology, Wuhan, Hubei province, China; Kaplan Medical Center, ISRAEL

## Abstract

Non-pharmacological therapies, especially the physical maneuvers, are viewed as important and promising strategies for reducing syncope recurrences in vasovagal syncope (VVS) patients. We observed the efficacy of a modified Valsalva maneuver (MVM) in VVS patients. 72 VVS patients with syncope history and positive head-up tilt table testing (HUTT) results were randomly divided into conventional treatment group (NVM group, n = 36) and conventional treatment plus standard MVM for 30 days group (MVM group, n = 36). Incidence of recurrent syncope after 12 months (6.5% vs. 41.2%, P<0.01) and rate of positive HUTT after 30 days (9.7% vs.79.4%, P<0.01) were significantly lower in MVM group than in NVM group. HRV results showed that low frequency (LF), LF/ high frequency (HF), standard deviation of NN intervals (SDNN) and standard deviation of all 5-min average NN intervals (SDANN) values were significantly lower in the NVM and MVM groups than in the control group at baseline. After 30 days treatment, LF, LF/HF, SDNN, SDANN values were significantly higher compared to baseline in MVM group. Results of Cox proportional hazard model showed that higher SDNN and SDANN values at 30 days after intervention were protective factors, while positive HUTT at 30 days after intervention was risk factor for recurrent syncope. Our results indicate that 30 days MVM intervention could effectively reduce the incidence of recurrent syncope up to 12 months in VVS patients, possibly through improving sympathetic function of VVS patients.

## Introduction

Vasovagal syncope (VVS) is a clinical syndrome resulting from systemic hypotension due to transient global cerebral hypoperfusion, and characterized by rapid onset, short duration and spontaneous complete recovery [1]. There are three types of VVS: vasodepressor syncope, cardio-inhibitory syncope and mixed syncope. Although not directly responsible for increased mortality, VVS could have a tremendous deleterious impact on the daily quality of life of patients in terms of physical symptoms and injury as well as psychological impact from living in fear of the next syncopal episode [2–4].

Therapeutic options for VVS remain challenging and far from optimal now [5–7]. Nowadays, following strategies were attempted to reduce syncope recurrences in VVS patients with variable efficacies: 1) physical techniques to improve orthostatic tolerance; 2) pharmacologic interventions to prevent depletion of intravascular volume and/or enhance arterial and venous tone; 3) cardiac pacing to avert bradycardia [4,8]; and 4) anatomically guided endocardial catheter ablation of ganglionated plexi in left atrium [9]. Among above mentioned therapy options, non-pharmacological therapies, especially the physical maneuvers, are viewed as important and promising strategies for reducing the syncope recurrences [5]. In a previous study, Krediet and colleagues demonstrated beneficial effects of leg crossing and muscle tensing in VVS patients [10]. Brignole et al. also reported a comparable effect of isometric arm counterpressure maneuvers to abort impending VVS [11]. In another study, van Dijk et al. showed that physical counterpressure maneuvers were effective in preventing vasovagal syncope [12].

Head-up tilt testing (HUTT) is a usual medical procedure to diagnose VVS and to define potential causes of syncope. Previous report showed that Valsalva maneuver (VM) could induce syncope in volunteer subjects [13], and the abrupt reduction in mean arterial blood pressure and subsequent reduction of cerebral perfusion during phase III of VM might be responsible for VM-induced syncope [14]. Based on these reports, we once asked patients with suspected syncope to perform the classic VM for several times at the beginning and during HUTT in an attempt to increase the positive rate of HUTT in these patients. To our surprise, post VM, the HUTT result became negative in 4 out of 8 patients with previous positive HUTT results. This finding encouraged us to test if VM could have therapeutic effects in syncope patients, and two aged (>65 years) hospitalized male patients with recurrent in-hospital syncope was instructed to perform the classical VM 2 times per day for 5 days under supervision of medical staffs, no syncope occurred during the subsequent hospital stay and these two patients were discharged after 1 week and were asked to perform the VM once daily for 30 days at home, syncope did not occur thereafter. In spired by above observations and we started a literature search to find out if there was a potential link between VM and VVS [15]. Quantified the individual roles of the cardiovascular system and the autonomic nervous system (ANS) in the hemodynamic regulations during the VM and found that hemodynamic responses to the VM were not only determined by the ANS-mediated cardiovascular regulations, but also significantly affected by the postural-change-induced hemodynamic alterations preceding the VM. We therefore assumed that the beneficial effects of VM observed in above circumferences might be related to the modulating role of VM on autonomic nervous function of VVS patients and we therefore designed present study to test if VM could be an eligible therapeutic option for VVS patients and observed the autonomic nervous function change post VM in VVS patients. To reduce the likelihood of inducing syncope by classical VM, a modified step-up graded VM (MVM) was proposed and patients were trained to perform the MVM in two phases: accommodation phase (≤ 10 days) and 30 days standard therapy phase with fixed time and strength schedule. Incidences of positive HUTT and recurrent syncope were compared in VVS patients with or without MVM intervention.

## Materials and methods

### Study population

**Fig 1:**
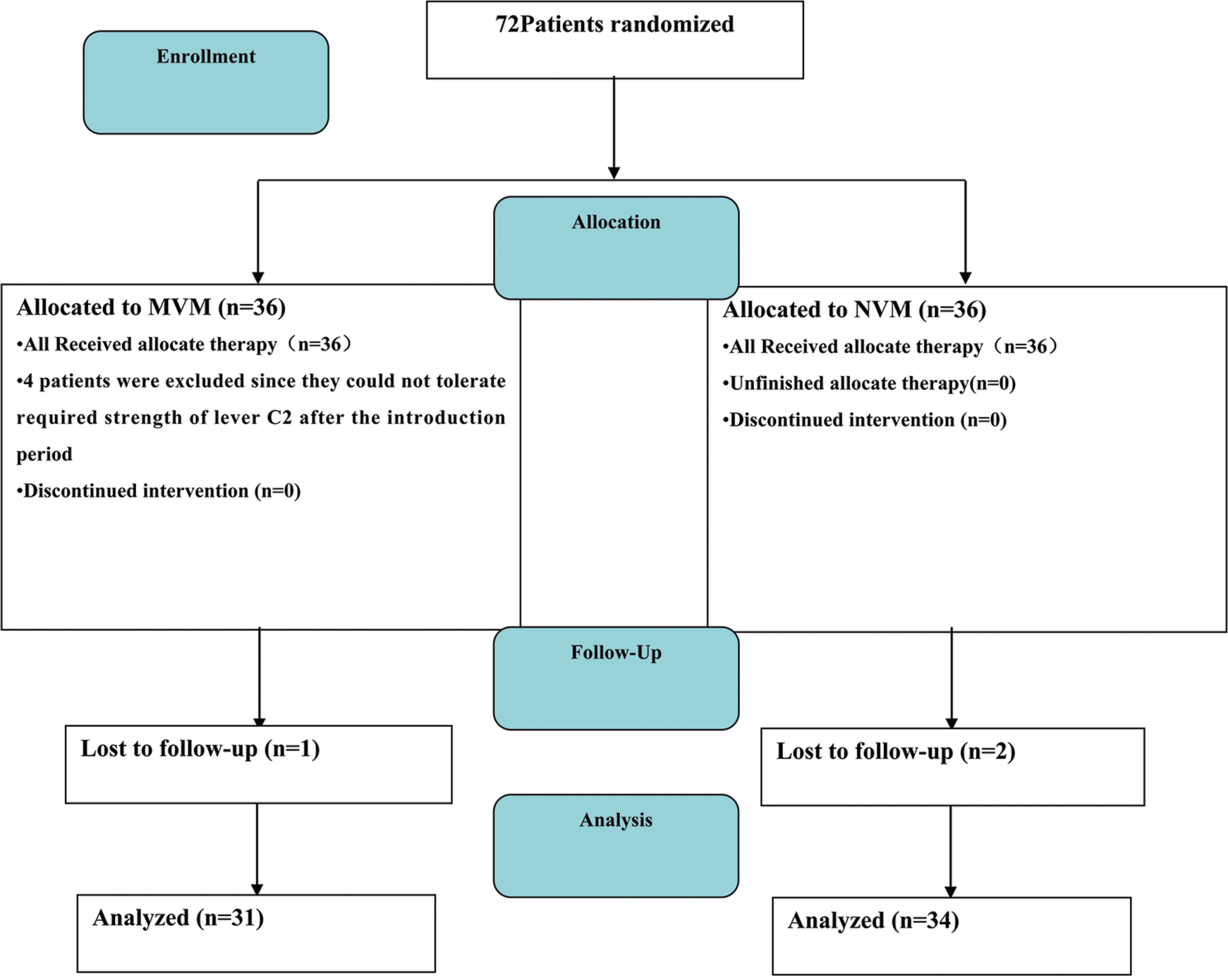
Diagram of patient flow. Consolidated reporting standard for trials diagram of patient flow.

Adult (aged between 18 to 80 years old) patients with recurrent VVS and recognizable prodromal symptoms were eligible for inclusion in our hospital. The diagnosis of VVS was based on the definition of the European Society of Cardiology guidelines [6]. Briefly, the diagnosis was considered certain with a typical history with episodes triggered by prolonged upright position, pain, or emotional events and accompanied by lightheadedness, sweating, pallor, and/or nausea/vomiting. Presyncope was defined as near loss of consciousness. From October 2012 through May 2015, we recruited patients with at least three lifetime syncopal spells associated with positive HUTT, and ≥ 1 syncope recurrence within 2 weeks and follow-up for one year after 30 days treatment. Patients with other causes of syncope, such as cardiac, cerebral, pulmonary, and metabolic diseases, were excluded by medical history, 12-lead ECG, skull CT scanning, chest x-ray, echocardiography and routine laboratory tests. Patients suffered from serious liver and renal diseases as well as moderate and severe anemia were excluded from this study (Biochemical parameters were listed in Table 1). Finally, 72 consecutive patients with recurrent VVS and positive HUTT test were recruited and randomly divided into MVM and NVM groups by biased coin design randomization method[16] (n = 36 each. We planned to recruit 30 for each group in our trial protocol. During the trial period, we recruited 36 patients for each group to ensure the required statistical power in case of loss of patients). (Fig 1). Conventional therapy included: 1) patient education for the pathophysiology and the benign nature of VVS and the importance of taking enough dietary salt and fluid in daily life; 2) avoiding medications which might precede VVS, like diuretics, and vasodilators. Thirty age and gender matched healthy volunteers were enrolled as normal controls. Twenty-four-hour Holter monitoring was performed before HUTT test and after 30 days standard MVM or NVM treatment. All patients gave informed consent for participation in this study, and written consent was obtained from each participating patient. The study protocol was approved by the ethical committees of Puai Hospital, Huazhong University of Science and Technology, Wuhan, China. The study was registered in the Chinese Clinical Trial Register (http://www.chictr.org.cn, registration number: ChiCTR-TRC-12002514) in September 12, 2012.

**Table 1:** Baseline characteristics of three groups.

	Control (n = 30)	NVM (n = 34)	MVM (n = 31)
Age (years)	56.1±17.7	58.4±16.3	54.8±14.4
Female (n)	17	20	18
Body mass index (kg/m^2^)	24.8±2.6	23.8±2.6	24.4±2.7
Supine systolic blood pressure (mmHg)	118±14	121±16	123±17
Supine diastolic blood pressure (mmHg)	70±7	73±9	71±8
Supine heart rate (bpm)	72±9	74±10	70±8
Syncope history			
Lifetime number of spells	0	6.4±4.5	7.2±6.4
Number of spells in the previous year	0	2.1±1.4	2.4±1.4
Duration of symptoms (years)	0	9.4±5.7	10.3±10.4
Trauma due to syncope (%)	0	14.7%	12.9%
Syncope type (n)			
Vasodepressor syncope	0	12	10
Cardio-inhibitory syncope	0	1	2
Mixed syncope	0	21	19
Biochemical parameters			
GOT (U/L)	29.8±13.9	28.4±12.6	25.0±12.7
GPT (U/L)	29.3±13.7	26.2±11.1	27.7±12.1
Hb (g/L)	132.3±11.3	129.9±12.3	127.8±13.9
Cr (umol/L)	64.5±14.4	71.4±15.8	69.1±17.5
Glu (mmol/L)	5.07±0.71	5.28±0.74	5.45±0.96
Disease history (n)			
Hypertension	0	5	4
Heart disease	0	3	3
Diabetes	0	1	2
Hyperlipidemia	0	6	7
Medications (%)			
CCB	0	8.8	9.7
BB	0	8.8	12.9
ACEI/ARB	0	11.8	9.7
Antidiabetic drugs	0	2.9	6.5
Lipid lowering drugs	0	17.6	19.4

NVM: patients treated with conventional therapy; MVM: patients treated with conventional therapy plus MVM for 30 days group; GPT: glutamate pyruvate transaminase; GOT: glutamic oxaloacetic transaminase; Cr: creatinine; Glu: glucose; Hb: hemoglobin; CCB: calcium channel blockers; BB: β-blocker; ACEI/ARB: angiotensin-converting enzyme inhibitors/angiotensin receptor blocker

### Twenty-four-hour Holter monitoring and HRV analysis

All recordings were performed with 24-hour Holter monitoring (GE Seer Light recording box and MARS Software) before HUTT at baseline and after 30 days in all participants. NN intervals was RR intervals in sinus rhythm and average NN intervals was the mean value of RR intervals in sinus rhythm. Quantitative heart rate variability (HRV) analysis was performed according to the guidelines of the European Society of Cardiology and the North American Society of Pacing and Electrophysiology [17]. HRV parameters were calculated in the time domain and frequency domain. Time domain related parameters included: standard deviation of NN intervals (SDNN), standard deviation of all 5-min average NN intervals (SDANN), square root of mean of the sum of squares of successive NN interval differences (rMSSD), number of successive NN interval differing by >50ms divided by the total number of successive NN intervals (pNN50). Frequency domain related parameters included: low frequency (LF) at frequency between 0.04–0.15 Hz, high frequency (HF) at frequency between 0.15–0.40 Hz, and the low frequency/high frequency ratio (LF/HF) [18].

### HUTT

Conventional HUTT was performed at baseline and after 30 days in all VVS patients. Electrocardiogram and systolic and diastolic blood pressure were continuously monitored and recorded. The patients were placed in the supine position for 10 minutes to obtain baseline ECG and blood pressure recordings. Patients were tilted to a 70° angle for 20 minutes. If syncope did not occur after 20 minutes, 300 mg nitroglycerin was administered sublingually, and the test was continued for another 15 minutes. If positive response occurred, HUTT was terminated immediately. HUTT would be deemed to be positive if syncope or presyncope occurred in association with hypotension (systolic pressure ≤80 mm Hg, diastolic pressure ≤50 mm Hg, or mean arterial pressure decrease ≥25%), and/or cardiac arrhythmia including sinus bradycardia ≤40 bpm, repetitive sinoatrial block or sinus pause >3 seconds, or Mobitz II 2nd or 3rd degree atrioventricular block.

### MVM protocols

#### Rational for the modification of the VM and detailed procedures of the MVM

**Fig 2:**
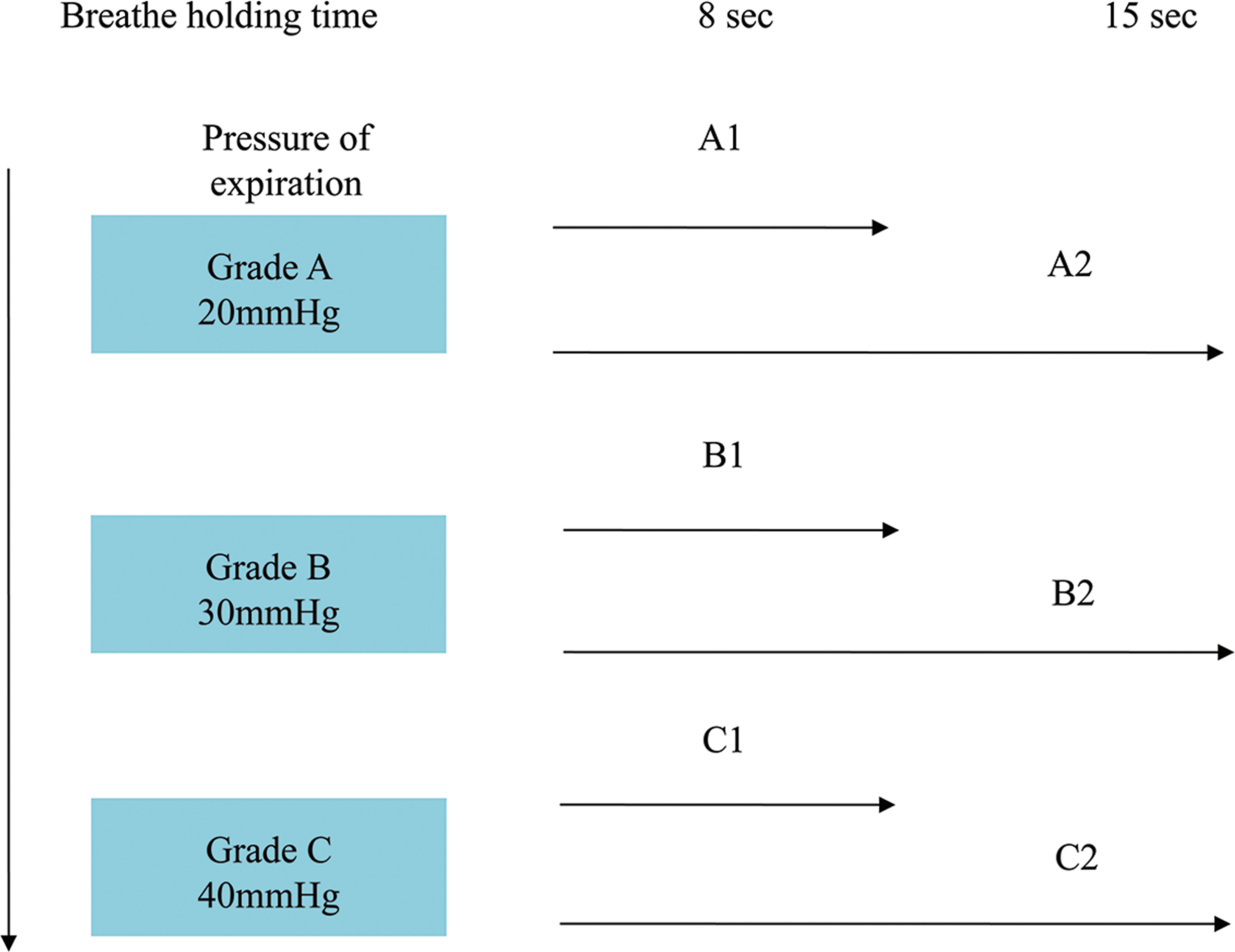
VM threshold level. Detailed procedures of the MVM for expiration strength grades and breath-hold time.

Previous report indicated that classic VM might induce syncope due to reduced venous return and decline in systolic blood pressure [13]. To reduce the likelihood of inducing syncope by VM, we designed a MVM procedure to increase the toleration of VM without increasing the potential risk of syncope for VVS patients, VM was graded by expiration strength (mm Hg) and breath-hold time (second). Expiration strength was divided into 3 grades (Grade A = 20 mm Hg, Grade B = 30 mm Hg and Grade C = 40 mm Hg) and breath-hold time was divided into 2 lengths (1 = 8 seconds, 2 = 15 seconds) (Fig 2). Expiration strength was controlled by blowing into a tube connected to sphygmomanometer.

In detail, MVM included three steps: 1. determine the threshold VM level; 2. accommodation phase: step-up graded VM began with tolerated level of VM till reaching the level C2 (Fig 2); 3. standardized modified VM therapy phase with fixed time and strength schedule (15 VMs at C2 level/day for 30 days).

#### Threshold of tolerated level of VM determination

To determine the tolerated level of VM of individual patient assigned to MVM group, patients were asked to perform VM in the order of A1, A2, B1, B2, C1 and C2 level. The VM level, which could be well tolerated without significant blood pressure and heart rate changes, was defined as threshold VM level for each individual patient.

#### Accommodation phase

- During the accommodation phase, the maneuvers were performed in 2 sessions every day (one in the morning and one in the afternoon, morning session consisted 8 VM and afternoon session consisted 7 VM) under the supervision of research staff, beginning with one level lower than the previously determined threshold VM level.
- Patients were asked to perform VM at one level higher in case of tolerating the current level for two times till reaching the C2 level.
- This accommodation period should be finished within 10 days and patients who did not reach the C2 level within 10 days were excluded from the study.

#### Standardized MVM therapy phase

This phase is defined as the application of 15 MV at C2 level daily for 30 days. MVM at this stage could be performed at outpatient department or at patient’s home or office and patients were asked to fill the MVM record sheet.

#### Follow-up

All Patients were followed up 12 months by phone call or home/clinic visit. The main endpoint was recurrent syncope.

### Statistical analysis

Continuous variables were presented as mean ± standard deviation (SD). Normal distribution of continuous variables was performed using Kolmogorov-Smirnov test. The homogeneity of variance test was performed by Levene test. Continuous data with normal distribution were assessed by Student’s *t*-test or one-way ANOVA with Post Hoc test (Bonferroni) as indicated. Non-normal distribution data were tested by two-tailed Mann–Whitney U test or Kruskal-Wallis non-parametric test as indicated. The Chi-square test was used to compare the ratio or percentages of categorical variables. The rates of recurrent syncope of NVM and MVM groups were compared by log rank test of Kaplan-Meier curve. The relationship between HUTT, HRV and recurrent syncope in VVS patients was assessed by Cox proportional hazards model. Univariable Cox regression analysis was performed for all clinical parameters and HRV parameters after 30 days treatments and variables with P values of <0.1 were included in multivariable Cox regression models. The choice of confounders was based on following considerations: age and gender belonged to the basic parameters which should be adjusted and HRV could be significantly affected by beta-blockers. Receiver operating characteristic (ROC) curves of HRV values were used to predict the recurrent syncope. The cutoff values of HRV were derived from ROC curve analysis by maximizing the sum of the sensitivity and specificity. All statistical analyses were performed with SPSS version 22.0. P<0.05 was considered statistically significant.

## Results

### Baseline characteristics of the participants

Baseline characteristics of participants are shown in Table 1. Age, gender, heart rate and blood pressure were similar among three groups. Frequency, duration of syncope, syncope-induced trauma, syncope type as well as disease history and current medications were also similar between patients in MVM and NVM groups. Glutamate pyruvate transaminase (GPT), glutamic oxaloacetic transaminase (GOT), creatinine (Cr), blood glucose (Glu) and hemoglobin (Hb) levels were similar among three groups.

### VM threshold level

After the accommodation phase, 4 patients in MVM group were excluded from the study since they developed symptoms and signs of syncope at VM level below C2 (1 at B1 level, 2 at B2 level and 1 at C1 level). One patient in MVM group and 2 patients in NVM group lost to follow-up and 31 patients in MVM group and 34 patients in NVM group were finally analyzed.

### Positive rate of HUTT after 30 days treatment

**Fig 3:**
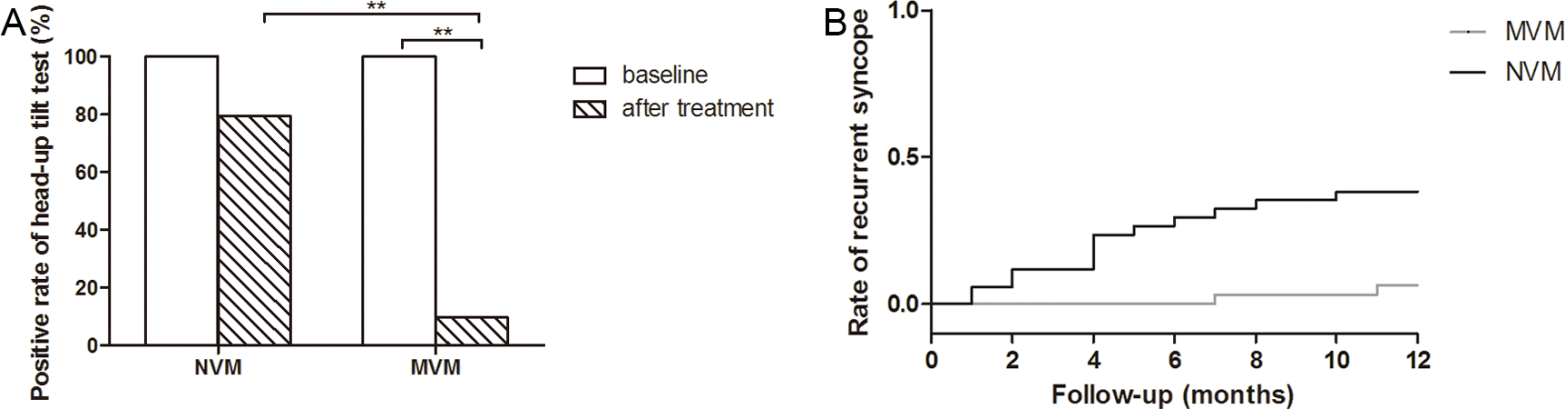
Positive rate of HUTT and the incidence of recurrent syncope in NVM and MVM group. **(**A) positive rate of HUTT at baseline and after 30 days treatment in NMV and MVM groups, (B) The Kaplan-Meier curves for recurrent syncope rates during 12 months follow-up in NMV and MVM groups. ** *P*<0.01.

HUTT was positive in all VVS patients at baseline. The percentage of positive conventional HUTT and provocative (nitroglycerin) HUTT was 9.7% and 90.3%, 8.8% and 91.2% respectively in MVM and NVM group at baseline. After 30 days treatment, the positive rate of HUTT was 9.7% in MVM group and 79.4% in NVM group (P<0.01, Fig 3A).

### Follow-up results

There were 2 patients with recurrent syncope in MVM group and 14 patients with recurrent syncope in NVM group during 12 months follow-up. There were only 2 vasodepressor syncope patients with recurrent syncope, and no cardio-inhibitory and mixed syncope patient with recurrent syncope in MVM group. There were 5 vasodepressor syncope patients and 9 mixed syncope patients with recurrent syncope, and no cardio-inhibitory syncope with recurrent syncope in NVM group during 12 months follow-up. Recurrent syncope of vasodepressor syncope was similar between MVM and NVM groups. Recurrent syncope of mix syncope was significantly reduced in MVM group compared to NVM group. During the follow-up period after therapy, syncope recurrence rate was 0% and 5.9% in the MVM and NVM group at one month, 0% and 23.6% at 3 months, 3.2% and 29.5% at 6 months and 6.5% and 41.2% at the 12 months. Kaplan-Meier curves suggested that the incidence of rate of recurrent syncope was significantly lower in the MVM group than in the NVM group (log rank test, *P* = 0.001, Fig 3B).

#### Relationship between HUTT at 30 days after intervention and incidence of recurrent syncope

**Fig 4:**
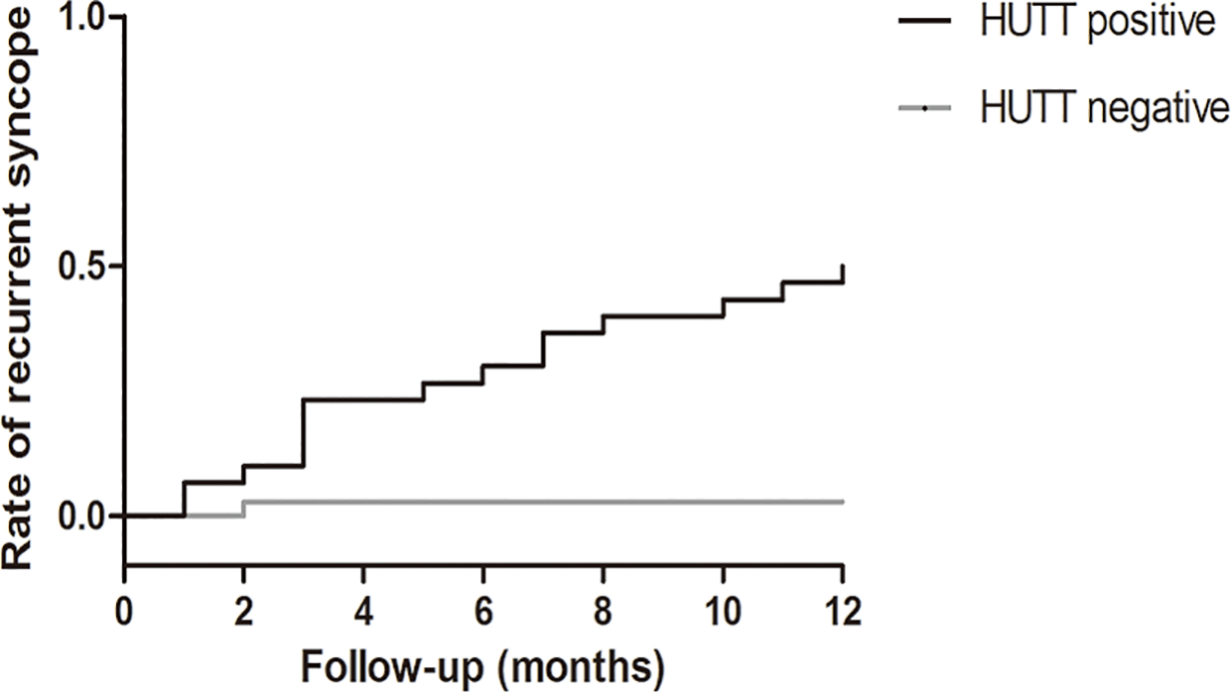
HUTT and the incidence of recurrent syncope in all VVS patients. The Kaplan-Meier curves for recurrent syncope rates during 12 months follow-up of patients from HUTT positive and HUTT negative groups.

All VVS patients were divided into HUTT negative group and HUTT positive group according to the HUTT results at 30 days post NVM and MVM intervention. There were 35 patients in HUTT negative group and 1 patient developed recurrent syncope during 12 months follow-up, and 30 patients in HUTT positive group and 15 patient developed recurrent syncope during 12 months follow-up. Kaplan-Meier curves suggested that the incidence of rate of recurrent syncope was significantly higher in the HUTT positive group than in the negative HUTT group (log rank test, *P* = 0.000, Fig 4). Results of Cox proportional Hazard model showed that positive HUTT at 30 days after intervention was a significant risk factor for recurrent syncope (HR = 22.38, 95%CI 2.95–169.85, *P* = 0.003, and the adjusted HR = 24.01, 95%CI 3.09–186.59, *P* = 0.002 after adjustment with gender, age and beta-blockers Table 2).

**Table 2:** Risk factors of recurrent syncope during the follow-up 12 months obtained from Cox proportional hazards models post 30 days intervention.

	unadjusted		adjusted gender, age and β-blockers	
	HR (95% CI)	*p* value	HR (95% CI)	p value
HUTT	22.38 (2.95–169.85)	0.003	24.01 (3.09–186.59)	0.002
SDNN	0.974 (0.951–0.997)	0.029	0.966 (0.937–0.996)	0.025
SDANN	0.966 (0.939–0.993)	0.014	0.950 (0.915–0.986)	0.007
LF/HF	0.159 (0.021–1.216)		0.278 (0.039–1.968)	0.200

SDNN: standard deviation of NN intervals; SDANN: standard deviation of all 5-min average NN intervals; LF: low frequency; LF/HF: low frequency/high frequency ratio.

#### Relationship between HRV at 30 days after intervention and incidence of recurrent syncope

**Fig 5:**
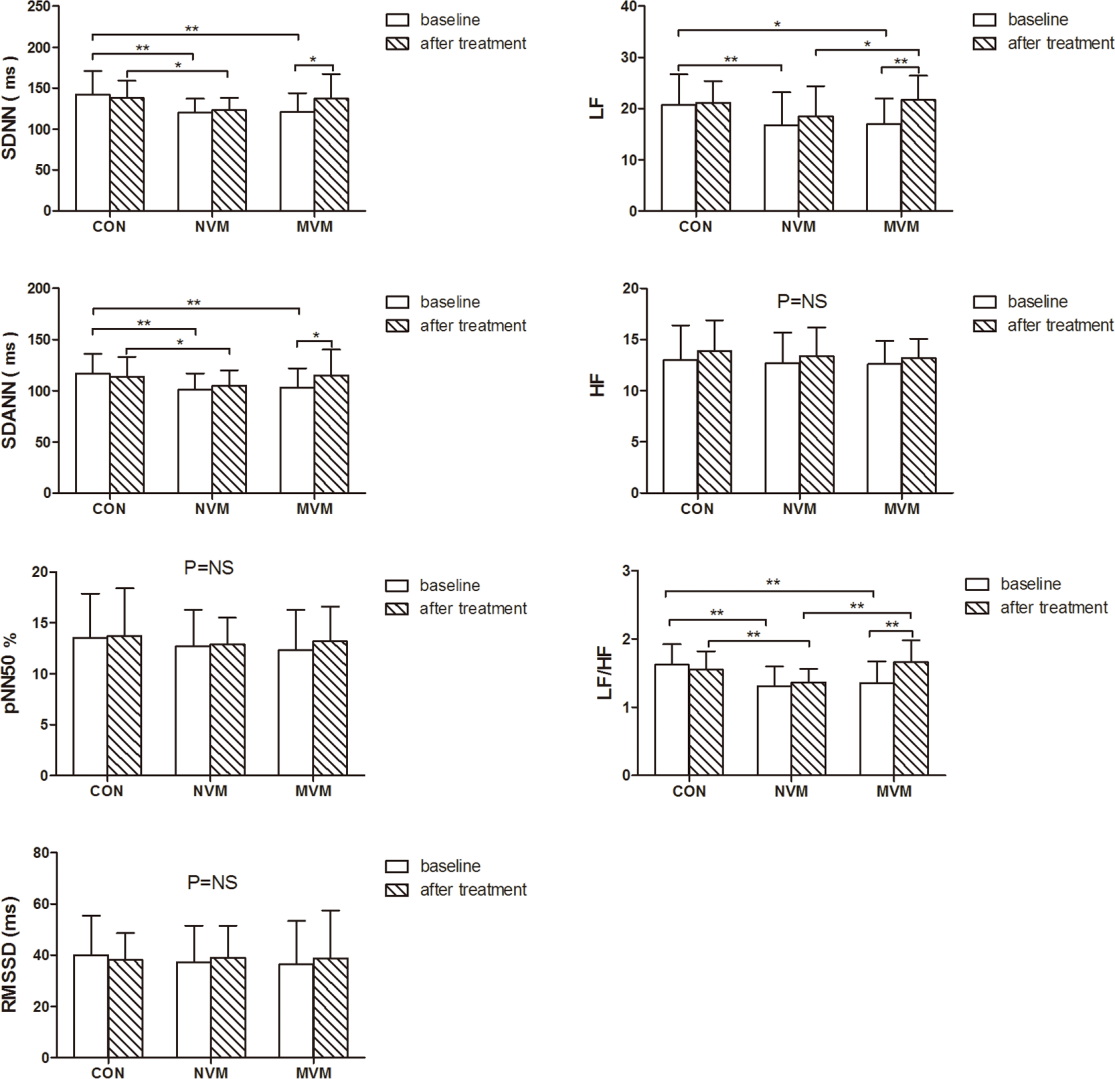
HRV analysis. HRV analysis of CON, NVM and MVM groups at baseline and after 30 days treatment. * *P*<0.05; ** *P*<0.01.

LF, LF/HF, SDNN and SDANN values were significantly lower in the NVM and MVM groups than in the control group at baseline. Average NN intervals, HF, rMSSD and pNN50 values were similar among the three groups at baseline and after 30 days treatment. LF and LF/HF values were significantly higher in MVM group than in NVM group after 30 days treatment. LF and LF/HF values were similar between MVM group and CON group after 30 days treatment. LF, LF/HF, SDNN and SDANN values were significantly higher compared to baseline in MVM group (Fig 5).

**Fig 6:**
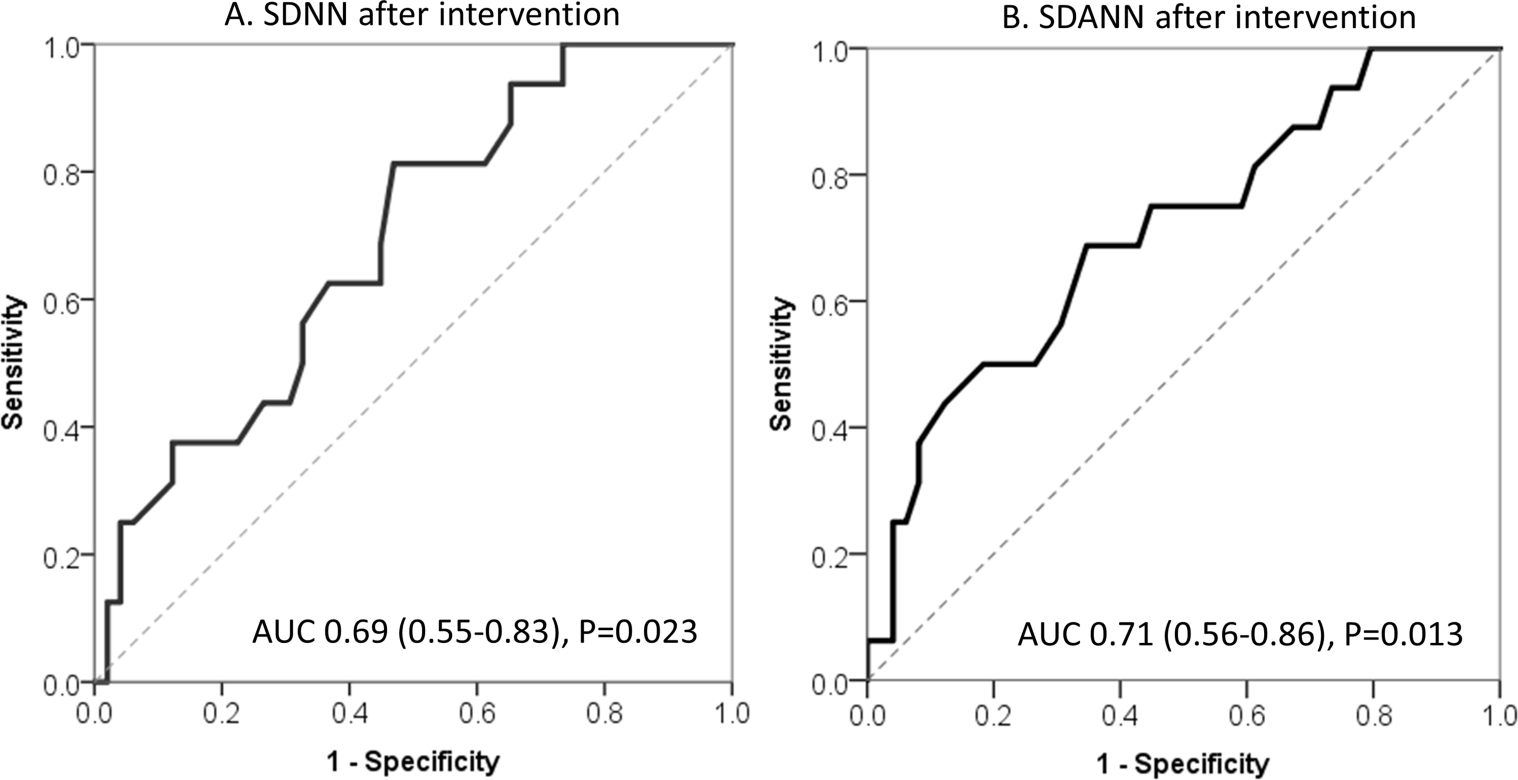
ROC curve of SDNN and SDANN. **(**A) ROC curve of SDNN after 30 days treatment, (B) ROC curve of SDANN after 30 days treatment.

Results of Cox proportional hazard model showed that higher SDNN and SDANN values, but not LF/HF ratio at 30 days after intervention, before and after adjusting for gender, age and beta-blockers, were protective factors for syncope recurrence (SDNN: HR = 0.974, 95%CI 0.951–0.997, *P* = 0.029; SDANN: HR = 0.966, 95%CI 0.939–0.993, *P* = 0.014; SDNN: adjusted HR = 0.966, 95%CI 0.937–0.996, *P* = 0.025; SDANN: adjusted HR = 0.950, 95%CI 0.915–0.986, *P* = 0.007, Table 2). The ROC curve analysis showed that the sensitivity and specificity for predicting no recurrent syncope were 53.1% and 81.2%, respectively, with a SDNN cutoff value ≥129 at 30 days after intervention, and the area under the curve was 0.690 (95%CI 0.549–0.831, *P =* 0.023 Fig 6A), and the sensitivity and specificity were 65.3% and 68.7%, respectively, with a SDANN cutoff value ≥103.5, and the area under the curve was 0.709 (95%CI 0.561–0.856, *P =* 0.013 Fig 6B)

## Discussions

Present study showed that the MVM intervention for 30 days is safe, feasible and effective to treat patients with VVS and MVM significantly reduced the positive rate of HUTT and recurrence rate of syncope. Moreover, LF, LF/HF, SDNN and SDANN values were all reduced in syncope patients compared to control subjects at baseline. HRV analysis showed that LF, LF/HF, SDNN and SDANN were significantly increased in patients allocated to MVM compared to baseline. Results of Cox proportional hazard model showed that higher SDNN and SDANN values at 30 days post intervention were protective factors, while positive HUTT was a risk factor for recurrent syncope. Autonomic nervous function is assumed to be normal during the non-syncope period of VVS patients [19]. However, our study results indicated that there was an abnormally increased sympathetic activity during the non-syncopal period of VVS patients. MVM could improve sympathetic function, which might be one potential mechanism responsible for the beneficial effects of MVM in VVS patients, especially for mix syncope patients. To our best knowledge, this is the first report on the efficacy and potential mechanism of MVM in VVS patients.

### Effects and compliance of modified VM in VVS patients

Till now, the therapy option for VVS is far away from optimal. Recently, several studies demonstrated satisfactory therapeutic results of physical training in VVS patients [10–12]. In our study, patients with recurrent VVS and positive HUTT test were recruited and randomly divided into MVM and NVM groups (Fig 1). Present study reported exciting short-term and long-term effectiveness of the MVM for VVS patients to reduce the positive rate of HUTT and recurrence rate of syncope. (Fig 3). Although repeated HUTT was not recommended to assess the therapeutic effects of various strategies in VVS patients [6], our results suggested positive HUTT was related significantly higher risk for recurrent syncope, indicating the value of repeated HUTT for predicting therapy efficacy in selected patients (Fig 4). Our study also demonstrated that recurrent syncope of mix syncope was significantly reduced in MVM group compared to NVM group during 12 months follow-up, which implied that mix syncope would benefit most from MVM treatment. Our results showed that MVM practiced in this study setting was safe, inexpensive and easy to perform, well tolerated and associated with good compliance (except one patient lost to follow up in MVM group, none of the rest patients in MVM group discontinued the MVM and none of the VVS patients in MVM group experienced syncope during the 30 days standard MVM intervention), therefore, MVM might serve as a feasible and effective therapeutic alternative option for VVS patients. Moreover, after the accommodation phase, which was performed under the supervision of medical staffs, this MVM was safely practiced by patients themselves at outpatient clinics, home or office during the standardized MVM phase. Above points might be responsible for the good compliance of MVM observed in this VVS patient cohort.

### Methodological viewpoint of the MVM

In our experience, the step-up procedure, in that the classic VM was divided into A1 to C2 levels (6 sub-levels, Fig 2), and patients were trained to increase the expiration strength and length of VM from A1 to C2 under the supervision of medical staffs during the around 10 days accommodation phase, then perform 15 MVM daily for another 30 days at C2 level, serves as the key points responsible for the satisfactory compliance and safety of MVM in this VVS patient cohort. In fact, except the unknown situation of the one lost to follow up patient, no patient experienced syncope during the MVM procedure. It is known that classical VM might “proceed” rather than treat VVS. In fact, the “begin low and go slow” principle applied in the present MVM works well in studied VVS patients, the gradually increased expiration strength and length of VM might therefore be crucial to avoid the unwanted impact of classical VM on hemodynamic and ANS in VVS patients.

### Potential mechanism of MVM on VVS

Although we observed satisfactory effects of MVM in studied VVS patients, the underlying mechanisms is not fully clear now and could not be proved with certain by present study, as fairly pointed out by Coffin et al [20]. VVS is now recognized as a disease related with autonomous neural disorder [18], and HRV is a commonly used method to evaluate the autonomic nervous function nowadays [21]. The mechanisms of VVS episode is characterized by increased cardiovagal tone and reduced peripheral sympathetic activity [7]. Autonomic nervous function is assumed to be normal during the non-syncope period of VVS patients [19]. Present results (Fig 5) found that HF, rMSSD and pNN50 (parameters reflecting the parasympathetic activities) were similar among control and syncope patients, suggesting the parasympathetic function was normal during the non-syncopal period of VVS patients. However, SDNN (reflecting total sympathetic and parasympathetic activity), LF (reflecting combined action of sympathetic and parasympathetic activity), LF/HF (reflecting the balance of sympathetic and parasympathetic function) and SDANN (reflecting sympathetic function) were all reduced in syncope patients compared to control subjects, indicating abnormally increased sympathetic activity in VVS patients during the non-syncopal period. LF and LF/HF values were similar between MVM group and CON group after 30 days treatment, indicating sympathetic activity returned to normal after 30 days MVM therapy. Previous studies showed that HRV results could be significantly affected by beta-blockers [22], our results showed that SDNN and SDANN values at 30 days post intervention were increased in VVS patients allocated to MVM group and results of Cox proportional hazard model indicated that higher SDNN and SDANN values at 30 days post intervention are protective factors for recurrent syncope, before and after adjusting for gender, age and beta-blockers use. Therefore, the difference in SDNN and SDANN values between VVS patients with NVM and MVM groups were unlikely induced by beta-blockers. SDNN ≥129 at 30 days post intervention was related to less syncope up to 12 months (Fig 6). Thus, our results might hint, to some extent, that the observed efficacy of MVM might at least partly be associated with the impact of MVM on autonomous nervous tone, in favor of improved sympathetic function, in VVS patients. Clearly, future studies comparing the effects of previously reported physical training [10–12] and present MVM in VVS patients and studies aiming to exploring the underlying working mechanisms of MVM are warranted.

### Limitations

Firstly, this is a single-center study with a small patient cohort. Studies with larger patient cohort are needed to validate the results of present study. Secondly, Holter monitoring and HRV analysis were not performed at 12 months after treatment to observe autonomic nervous function in VVS patients. Thirdly, the underlying working mechanisms of MVM in VVS remain largely unknown now and need to be explored in future studies.

## Conclusions

Our study results suggest that MVM therapy strategy is safe and associated with satisfactory short- and long-term effects in this VVS patient cohort. Our results further hint a role of improved sympathetic function in VVS post MVM. Future multi-center studies are warranted to validate results shown in the present study.

## Supporting information

S1 CONSORT Checklist CONSORT checklist. (DOC)

S1 Table Raw data of baseline characteristics. Raw data of all baseline characteristics for each patient in three groups.
(XLS)

S2 Table Raw data of HUTT. The raw data of HUTT for each patient in NVM and MVM groups at baseline and after 30 days treatment.
(XLS)

S3 Table Raw data of recurrent syncope incidence. The raw data of recurrent syncope incidence for each patient in NVM and MVM groups during 12 months follow-up.
(XLS)

S4 Table Raw data of HRV. HRV raw data of each patient in three groups at baseline and after 30 days treatment.
(XLS)

S1 Clinical Trial Protocol Clinical trial protocol in Chinese. (PDF)

S2 Clinical Trial Protocol Clinical trial protocol in English. (DOC)

S1 Ethics Committee Approval Document Ethics committee approval document in Chinese. (PDF)

S2 Ethics Committee Approval Document Ethics committee approval document in English. (DOCX)

## References

[1] Fenton AM, Hammill SC, Rea RF, Low PA, Shen WK. Vasovagal syncope. Ann Intern Med. 2000; 133: 714–725. 11074905 10.7326/0003-4819-133-9-200011070-00014

[2] Rose MS, Koshman ML, Spreng S, Sheldon R. The relationship between health-related quality of life and frequency of spells in patients with syncope. J Clin Epidemiol.2000; 53: 1209–1216. 11146266 10.1016/s0895-4356(00)00257-2

[3] Rose MS, Koshman ML, Ritchie D, Sheldon R. The development and preliminary validation of a scale measuring the impact of syncope on quality of life. Europace. 2009; 11: 1369–1374. doi: 10.1093/europace/eup106 19797151 10.1093/europace/eup106

[4] Benditt DG, Nguyen JT. Syncope: therapeutic approaches. J Am Coll Cardiol. 2009;53: 1741–1751. doi: 10.1016/j.jacc.2008.12.065 19422980 10.1016/j.jacc.2008.12.065

[5] Raj SR, Coffin ST. Medical therapy and physical maneuvers in the treatment of the vasovagal syncope and orthostatic hypotension. Prog Cardiovasc Dis. 2013; 55: 425–433. doi: 10.1016/j.pcad.2012.11.004 23472781 10.1016/j.pcad.2012.11.004 PMC3594734

[6] Moya A, Sutton R, Ammirati F, Blanc JJ, Brignole M, Dahm JB, et al Guidelines for the diagnosis and management of syncope (version 2009). Eur Heart J. 2009; 30: 2631–2671. doi: 10.1093/eurheartj/ehp298 19713422 10.1093/eurheartj/ehp298 PMC3295536

[7] Aydin MA, Salukhe TV, Wilke I, Willems S. Management and therapy of vasovagal syncope: A review. World J Cardiol. 2010; 2: 308–315. doi: 10.4330/wjc.v2.i10.308 21160608 10.4330/wjc.v2.i10.308 PMC2998831

[8] Sutton R. Should we treat severe vasovagal syncope with a pacemaker? J Intern Med. 2017; 3 14 doi: 10.1111/joim.12603 [Epub ahead of print] 28294432 10.1111/joim.12603

[9] Sun W, Zheng L, Qiao Y, Shi R, Hou B, Wu L, et al Catheter Ablation as a Treatment for Vasovagal Syncope: Long-Term Outcome of Endocardial Autonomic Modification of the Left Atrium. J Am Heart Assoc. 2016; 5: pii: e003471 doi: 10.1161/JAHA.116.003471 27402231 10.1161/JAHA.116.003471 PMC5015383

[10] Krediet CT, van Dijk N, Linzer M, van Lieshout JJ, Wieling W. Management of vasovagal syncope: controlling or aborting faints by leg crossing and muscle tensing. Circulation. 2002; 106: 1684–1689. 12270863 10.1161/01.cir.0000030939.12646.8f

[11] Brignole M, Croci F, Menozzi C, Solano A, Donateo P, Oddone D, et al Isometric arm counter-pressure maneuvers to abort impending vasovagal syncope. J Am Coll Cardiol. 2002; 40: 2053–2059. 12475469 10.1016/s0735-1097(02)02683-9

[12] van Dijk N, Quartieri F, Blanc JJ, Garcia-Civera R, Brignole M, Moya A, et al Effectiveness of physical counterpressure maneuvers in preventing vasovagal syncope: the Physical Counterpressure Manoeuvres Trial (PC-Trial). J Am Coll Cardiol. 2006; 48: 1652–1657. doi: 10.1016/j.jacc.2006.06.059 17045903 10.1016/j.jacc.2006.06.059

[13] Klein LJ, Saltzman HA, Heyman A, Sieker HO. Syncope Induced by the Valsalva Maneuver. A Study of the Effects of Arterial Blood Gas Tensions, Glucose Concentration and Blood Pressure. Am J Med. 1964; 37: 263–268. 14206758 10.1016/0002-9343(64)90010-5

[14] Duvoisin RC. Valsalva Maneuver in Study of Syncope. Electroencephalography and Clinical Neurophysiology. 1961; 13: 622–626. 13888763 10.1016/0013-4694(61)90178-x

[15] Liang F, Liu H. Simulation of hemodynamic responses to the valsalva maneuver: an integrative computational model of the cardiovascular system and the autonomic nervous system. J Physiol Sci. 2006; 56: 45–65. 16779913 10.2170/physiolsci.rp001305

[16] Sun Z, Braun TM. A two-dimensional biased coin design for dual-agent dose-finding trials. Clin Trials. 2015; 12: 596–607. doi: 10.1177/1740774515592404 26163309 10.1177/1740774515592404

[17] Heart rate variability: standards of measurement, physiological interpretation and clinical use. Task Force of the European Society of Cardiology and the North American Society of Pacing and Electrophysiology. Circulation. 1996; 93: 1043–1065. 8598068

[18] Sehra R, Hubbard JE, Straka SP, Fineberg NS, Engelstein ED, Zipes DP, et al Autonomic changes and heart rate variability in children with neurocardiac syncope. Pediatr Cardiol. 1999; 20: 242–247. doi: 10.1007/s002469900456 10368446 10.1007/s002469900456

[19] Virend K, Somers. Cardiovascular manifestations of autonomic disorders. In: Douglas LM, Douglas PZ, Peter L, Robert OB, editors. Braunwald’s heart disease;2015.10th edn, Philadelphia, PA, pp 1937.

[20] Coffin ST, Raj SR. Ongoing clinical trials for Vasovagal Syncope: where are we in 2014? Auton Neurosci. 2014; 184: 77–82. doi: 10.1016/j.autneu.2014.05.009 24913692 10.1016/j.autneu.2014.05.009 PMC4139437

[21] Sztajzel J. Heart rate variability: a noninvasive electrocardiographic method to measure the autonomic nervous system. Swiss Med Wkly. 2004; 134: 514–522. 15517504 10.4414/smw.2004.10321

[22] Jokinen V, Tapanainen JM, Seppanen T, Huikuri HV. Temporal changes and prognostic significance of measures of heart rate dynamics after acute myocardial infarction in the beta-blocking era. Am J Cardiol. 2003; 92: 907–912. 14556864 10.1016/s0002-9149(03)00968-8

