# Supplementary material for: A single-center randomized controlled trial observing the safety and efficacy of modified step-up graded Valsalva manoeuver in patients with vasovagal syncope
Source: PLoS One. 2018 Jan 30;13(1):e0191880. doi: 10.1371/journal.pone.0191880 (PMC5790265; doi:10.1371/journal.pone.0191880)
Supplement: S1 Clinical Trial Protocol — (PDF) [file pone.0191880.s006.pdf]

改良Valsalva动作对血管迷走性晕厥治疗效果的观察

（版本号 1.0 2012年8月20日）

普爱医院心内科

摘要

目前，迷走神经晕厥治疗的选择仍然充满挑战性。非药物治疗，特别是物理疗法，被认为是减少迷走神经晕厥再发的重要和具有前途的治疗方法。我们计划入选 60 例直立倾斜试验阳性的晕厥患者，随机分为常规治疗组（NVM 组，n = 30）和常规治疗加 30 天标准 MVM 治疗组（MVM 组，n = 30）。比较两组治疗 30 天后直立倾斜试验阳性率和 12 个月随访中晕厥再发情况；分析比较三组之间和每组基线与治疗 30 天后的心率变异性指标。通过观察改良的 Valsalva 动作对迷走神经晕厥患者治疗的安全性和有效性，我们试图寻找治疗迷走神经晕厥的新方法；并探讨迷走神经晕厥的发病机制，以及改良 Valsalva 动作治疗迷走神经晕厥的潜在机制。

研究背景及立题依据

迷走神经晕厥((VVS) 是一种常见的由于短暂性脑缺血引起全身性低血压反应的临床综合征。它具有突然、短暂的意识丧失，不能维持正常的直立体位，持续几秒钟至几分钟而自行完全恢复的特征[1]。尽管 VVS 不直接增加死亡率，但因患者害怕再发晕厥，从而增加身心负担，严重影响患者的生活质量[2-4]。VVS 的治疗是一个具有挑战性问题[5-6]，下列方法可能减少 VVS 患者再发晕厥：1）物理方法提高直立体位的耐受性；2）药物干预防止血容量突然下降；3）安装心脏起搏以避免心动过缓[4]。

直立倾斜试验（HUTT）是诊断 VVS 的常用方法。既往研究发现在自愿参加研究者中，Valsalva 动作（VM）能诱发晕厥[7]。我们要求疑似晕厥患者在 HUTT 开始和检查过程中作几次经典的 VM，以增加 HUTT 的诊断阳性率。让我们感到意外的是，以前 HUTT 阳性的 8 例患者，在检查中做了几个 VM 后，有 4 例患者 HUTT 转为阴性。这个发现使我们想到 VM 可能对 VVS 患者有治疗作用。在医务人员的监督下，我们教两个大于 65 岁因反复发作晕厥住院的男性患者每日进行 2 次经典的 VM 治疗，持续 5 天，这两个患者在住院期间没有再发晕厥，他们 1 周后出院。出院后，我们要求他们每日在家里继续坚持做 1 次 VM 治疗，持续 30 天，到目前为止，这两个患者已经大约 3 个月没有再发晕厥了。

目前认为，在迷走神经晕厥期，由于静脉回流减少，心脏前负荷下降，首先

出现交感神经激活，反射性触发迷走神经功能活性增加，导致心率下降和/或血压降低。我们认为 VM 对 VVS 患者的治疗作用，可能与 VM 对自主神经功能的调节有关[8]。心率变异性是评价自主神经功能的常用指标。为此，我们设计了这个研究方案，观察 VM 是否适合治疗 VVS 患者，并通过心率变异性分析，观察 VVS 患者自主神经功能的变化。为了减少经典 VM 诱发晕厥可能，我们采用逐步增加压力和时间的改良 VM 治疗 VVS 患者。

研究设计

前瞻性随机对照试验。

研究目的

1. 观察改良 Valsalva 动作治疗对 VVS 患者的疗效，寻找治疗 VVS 的新方法。
2. 探讨 VVS 的发病机制以及改良的 Valsalva 动作治疗 VVS 的潜在机制。

实验方法

1. 研究对象

入选标准：VVS 患者，年龄 18-80 岁之间，晕厥三次以上，倾斜试验阳性。

排除标准： 心、脑、肺及代谢疾病引起的晕厥；采用其它药物治疗的患者；正参加其它临床研究的患者；其他严重疾病及精神病患者；妊娠、准备妊娠和哺乳期妇女；拒绝参加该研究者。

2. 实验分组

VVS 患者随机分为两组，VVS 患者常规治疗组（NVM 组）和 VVS 患者 Valsalva 动作治疗组（MVM 组），每组 30 人。健康对照组（CON 组）30 人。

2.1 NVM 组：常规治疗包括：1)对患者进行健康教育，告知每日摄取足够水盐的重要性。2)避免使用利尿剂，血管扩张剂等药物。

2.2 MVM 组：在常规治疗的基础上，增加改良 Valsalva 动作每日一次，每次 50-60 分钟。

2.3 CON 组：入选无晕厥史的健康者为对照组，性别、年龄与试验组（NVM 组和 MVM 组）相匹配。

3　实验方法

所有 VVS 患者签署书面知情同意书后进行临床试验。试验前，记录三组患者基线状态下的年龄、性别、体重指数、心率、血压和生化指标，记录迷走神经晕厥患者晕厥的频率、持续时间、晕厥引起的创伤以及既往史和目前用药。

3.1 倾斜试验

所有 VVS 患者在实验开始基线状态和治疗 30 天结束后各做一次倾斜试验。

患者安静平卧倾斜试验床 10 分钟，记录基线状态下心率和血压，试验过程中连续监测心率和血压变化。患者直立倾斜至 70°，观察 20 分钟。如不出现阳性反应，舌下含服 0.3 mg 硝酸甘油继续观察 15 分钟。观察过程中如出现阳性反应，立即终止试验。患者出现血压下降(收缩压≤80mmHg 和/或舒张压≤50mmHg，或平均动脉压下降>25%)和/或心律失常(窦性心动过缓≤40 次/分，窦房阻滞或窦性停搏伴长达 3s 以上的心脏停搏，莫氏二度或三度房室阻滞)伴晕厥或晕厥前反应者，倾斜试验结果判断为阳性。

3.2　24小时动态心电监测

实验组开始倾斜试验前的基线状态和治疗30天结束后各做一次，观察时域和频域指标。

时域指标：

1）SDNN：即全部正常窦性心搏间期的标准差

2）SDANN：即24 h 内5分钟节段平均心动周期的标准差

3）rMSSD：即相邻正常心动周期差值的均方根

4）pNN50 (%)：即相邻正常窦性心搏间期差值> 50 ms 的心搏数占全部正常窦性心搏间期的比例

频域指标：

1）低频功率(LF)：频段0.04～0.15 Hz

2）高频功率(HF)：频段0.15～0.4 Hz

3）低频与高频之比(LF/HF)

3.3 改良Valsalva动作具体操作

3.3.1 改良 Valsalva 动作的呼气压力和屏气时间。患者呼气压力分为三个级别(A 级= 20 mm Hg，B 级= 30 mm Hg，C 级= 40 mm Hg)，呼气压力通过管道连接到血压计测定。屏气时间分为两种时长(1 = 8 秒, 2 = 15 秒)。

3.3.2 改良 Valsalva 动作三个阶段

3.3.2.1 阈值测定 让患者依次在 A1, A2, B1, B2, C1 和 C2 各水平做 Valsalva 动作，以患者能够耐受，不出现血压和心率的重大变化为个体的阈值水平。

3.3.2.2 导入期 让患者早上做 8 个 Valsalva 动作和下午做 7 个 Valsalva 动作。在能耐受的情况下，逐渐从 A1 水平升至 C2 水平。如果 10 天内患者达不到 C2 水平，将会被排除。

3.3.2.3 标准治疗期 让患者每天在 C2 水平做 15 个 Valsalva 动作，一次性完成，需要 50-60 分钟，坚持 30 天。

3.4 随访 标准治疗期 30 天完成后，随访一年，观察再次发生晕厥的时间。

统计学分析

连续变量采用均数 ±标准差表示。三组基线资料中，正态分布资料采用 Student’s *t* 检验或单因素方差分析（Bonferroni），非正态分布资料采用双尾 Mann–Whitney U 检验或 Kruskal-Wallis 非参数分析；分类变量中率或百分比的比较采用卡方检验。NVM 组和 MVM 组基线状态和治疗 30 天后的倾斜试验阳性率比较采用卡方检验。NVM 组和 MVM 组在 12 个月随访中，再发晕厥以 Kaplan-Meier 曲线表示，采用 log rank 检验分析。三组心率变异性指标比较中，正态分布资料采用单因素方差分析（Bonferroni），非正态分布资料用 Kruskal-Wallis 非参数分析。30 天治疗前后 HRV 各指标比较，正态分布资料采用 Student’s *t* 检验，非正态分布资料采用双尾 Mann–Whitney U 检验。30 天治疗后，迷走神经晕厥患者倾斜试验与 12 个月随访中再发晕厥之间的关系，心率变异性各指标与 12 个月随访中再发晕厥之间的关系，采用单变量和多变量 Cox 比例风险模型分析。治疗 30 天后，采用心率变异性各指标的 ROC（Receiver operating characteristic） 曲线，预测迷走神经晕厥患者 12 个月随访中再发晕厥，心率变

异性各指标的 cutoff 值以敏感性和特异性之和最大来获取。所有统计分析采用 SPSS 19.0 统计软件，以 P<0.05 为有统计学意义。

预期结果

CON 组，NVM 组和 MVM 组基线资料无统计学差异。30 天治疗完成后，再次做 HUTT，MVM 组 HUTT 阳性率低于 NVM 组；进行 12 个月随访，MVM 组再发晕厥率低于 NVM 组。通过对三组之间及各组基线水平和治疗后心率变异性分析提示，VVS 患者存在自主神经功能失调，改良 Valsalva 动作治疗 VVS 的潜在机制是改善自主神经功能。

技术路线图和工作计划

技术路线图

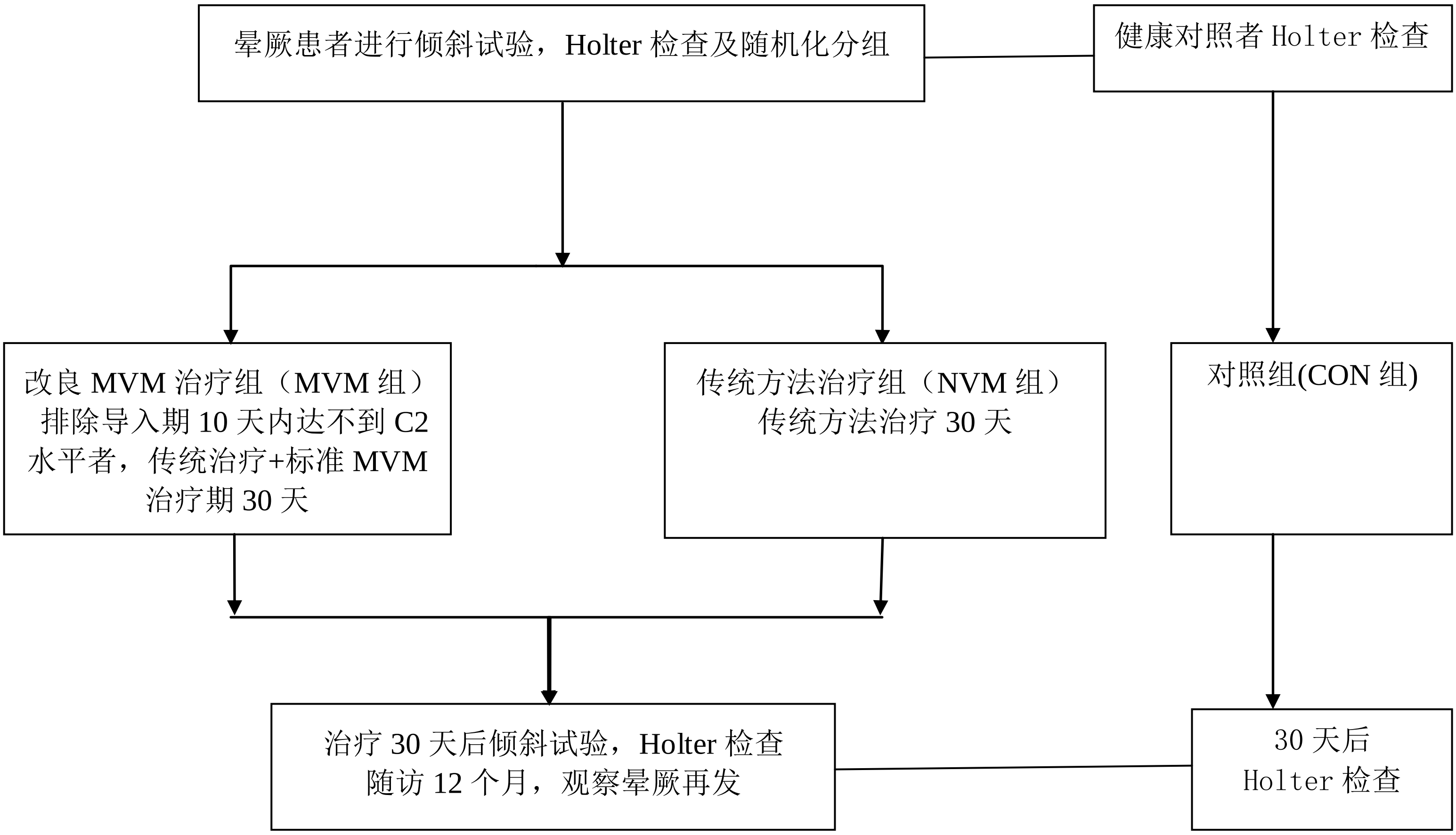

工作计划

1. 2012 年 5 月-2012 年 9 月　临床实验方案制定，伦理学审查和临床试验网上登记。

2. 2012 年 10 月-2014 年 12 月　临床病例入选，试验分组，试验过程，一年随访

3. 2015 年 1 月- 2015 年 6 月　试验数据资料统计分析和论文写作

4. 2015 年 7 月-　论文投稿和发表

参考文献

1. Fenton AM, Hammill SC, Rea RF, Low PA, Shen WK. Vasovagal syncope. Ann Intern Med. 2000; 133: 714-725.
2. Rose MS, Koshman ML, Spreng S, Sheldon R. The relationship between health-related quality of life and frequency of spells in patients with syncope. J Clin Epidemiol.2000; 53: 1209-1216.
3. Rose MS, Koshman ML, Ritchie D, Sheldon R. The development and preliminary validation of a scale measuring the impact of syncope on quality of life. Europace. 2009; 11: 1369-1374.
4. Benditt DG, Nguyen JT. Syncope: therapeutic approaches. J Am Coll Cardiol. 2009;53: 1741-1751.
5. Moya A, Sutton R, Ammirati F, Blanc JJ, Brignole M, et al. Guidelines for the diagnosis and management of syncope (version 2009). Eur Heart J. 2009; 30: 2631-2671.
6. Aydin MA, Salukhe TV, Wilke I, Willems S. Management and therapy of vasovagal syncope: A review. World J Cardiol. 2010; 2: 308-315.
7. Klein LJ, Saltzman HA, Heyman A, Sieker HO. Syncope Induced by the Valsalva Maneuver. A Study of the Effects of Arterial Blood Gas Tensions, Glucose Concentration and Blood Pressure. Am J Med. 1964; 37: 263-268.
8. Liang F, Liu H. Simulation of hemodynamic responses to the valsalva maneuver: an integrative computational model of the cardiovascular system and the autonomic nervous system. J Physiol Sci. 2006; 56: 45-65.
