# Supplementary material for: A single-center randomized controlled trial observing the safety and efficacy of modified step-up graded Valsalva manoeuver in patients with vasovagal syncope
Source: PLoS One. 2018 Jan 30;13(1):e0191880. doi: 10.1371/journal.pone.0191880 (PMC5790265; doi:10.1371/journal.pone.0191880)
Supplement: S2 Clinical Trial Protocol — (DOC) [file pone.0191880.s007.doc]

**The safety and efficacy of modified step-up graded Valsalva manoeuver in patients with vasovagal syncope**

**(Version 1.0 August 20, 2012)**

**Department of Cardiology Puai Hospital**

**Abstract**

Therapeutic options for vasovagal syncope (VVS) remain challenging now. Non-pharmacological therapies, especially the physical maneuvers, are viewed as important and promising strategies for reducing the syncope recurrences. We will plan to recruit 60 VVS patients with syncope history and positive head-up tilt table testing (HUTT). They will be randomly divided into conventional treatment group (NVM group, n=30) and conventional treatment plus standard MVM for 30 days group (MVM group, n=30). The rate of positive HUTT after 30 days treatment and incidence of recurrent syncope during 12 months follow-up will be observed between the two groups. Heart rate variability (HRV) indexes among the three groups and between baseline and after 30 days treatment will be analyzed. The safety and efficacy of the modified Valsalva maneuver (MVM) in VVS patients will be observed. We attempt to find out a new way to treat VVS and discovery the pathogenesis of VVS and the potential mechanism of MVM in VVS.

**Background**

Vasovagal syncope (VVS) is a common clinical syndrome resulting from systemic hypotension due to transient global cerebral hypoperfusion. It characterizes by rapid, brief loss of consciousness that cannot maintain a normal upright position and lasts for a few seconds to a few minutes and spontaneous complete recovery. Although not directly responsible for increased mortality, VVS has a tremendous deleterious impact on the daily quality of life of patients in terms of physical symptoms and injury as well as psychological impact from living in fear of the next syncopal episode . Therapeutic options for VVS remain a challenging problem . Following methods are attempted to reduce syncope recurrences in VVS patients: 1) physical techniques to improve orthostatic tolerance; 2) pharmacologic interventions to prevent depletion of intravascular volume; 3) cardiac pacing to avert bradycardia .

Head-up tilt testing (HUTT) is a usual procedure to diagnose VVS. Previous report showed that VM could induce syncope in volunteer subjects [7]. We asked patients with suspected syncope to perform the classic VM for several times at the beginning and during HUTT in an attempt to increase the positive rate of HUTT in these patients. To our surprise, the HUTT result became negative in 4 out of 8 patients with previous positive HUTT results after VM. This finding encouraged us to test if VM has therapeutic effects in VVS patients. We selected two aged (>65 years) hospitalized male patients with recurrent in-hospital syncope and taught them to perform the classical VM 2 times per day for 5 days under supervision of medical staffs. No syncope occurred during the subsequent hospital stay and the two patients were discharged after 1 week. We asked them to perform the VM once daily for 30 days at home. Until now, they have not occurred syncope for about 3 months.

It is considered that sympathetic activity increased with reduction of venous return and decrease of cardiac preload in the syncopal period of VVS patients at first, and reflex activity of vagus nerve led to the decrease of heart rate and / or blood pressure at present. We think that the therapeutic effect of VM in VVS patients may be related to the regulation of autonomic nervous function [8]. HRV is a common index to evaluate autonomic function. We design the study to test if VM can be an eligible therapeutic option for VVS patients and observe the autonomic nervous function change by HRV indexes in VVS patients. To reduce the likelihood of inducing syncope by classical VM, a modified step-up graded VM (MVM) will be proposed to treat VVS patients by increasing expiration strength and breath-hold time gradually.

**Study design**

Prospective randomized controlled study.

**Objective**

1. To observe the safety and efficacy of a modified Valsalva maneuver (MVM) in VVS patients and find out a new way to treat VVS.

2. To discovery the pathogenesis of VVS and the potential mechanism of MVM on VVS.

**Methods**

1. Study population

Inclusion criteria: we will recruit VVS patients aged between 18 to 80 years old with at least three lifetime syncopal spells associated with positive head-up tilt testing.

Exclusion criteria: Patients will be excluded if they have other causes of syncope, cannot give informed consent, have serious diseases or psychopath. Women who are pregnant, ready to pregnant, or breast-feeding a baby will be excluded.

2. Divided groups

VVS patients will be randomly divided into two groups: NVM group and MVM group, each 30 patients. Healthy control group (CON group) is 30 patients.

2.1 NVM group: Conventional therapy included: 1) Patient education for the importance of taking enough dietary salt and fluid in daily life. 2) Avoid medications such as diuretics and vasodilators.

2.2 MVM group: conventional treatment plus standard MVM for 50-60 minutes once a day for 30 days.

2.3 CON group: Age and gender matched healthy volunteers without syncope will be enrolled as healthy controls.

3. Study methods

All patients will give informed consent for participation in the study. Age, gender, body mass index, heart rate, blood pressure and biochemical indexes will be record at baseline in the three groups. Frequency and duration of syncope, syncope-induced trauma as well as disease history and current medications will be record in VVS patients.

3.1 Head-up tilt testing (HUTT)

HUTT will be performed at baseline and after 30 days in all VVS patients.

The patients are placed in the supine position for 10 minutes to obtain baseline ECG and blood pressure recordings. Electrocardiogram and systolic and diastolic blood pressure are continuously monitored and record. Patients are tilted to a 70° angle for 20 minutes. If syncope do not occur after 20 minutes, 300 mg nitroglycerin is administered sublingually, and the test is continued for another 15 minutes. If positive response occurs, HUTT is terminated immediately. HUTT will be deemed to be positive if syncope or presyncope occurs in association with hypotension (systolic pressure ≤80 mm Hg, diastolic pressure ≤50 mm Hg, or mean arterial pressure decrease ≥25%), and/or cardiac arrhythmia including sinus bradycardia ≤40 bpm, repetitive sinoatrial block or sinus pause >3 seconds, or Mobitz II 2nd or 3rd degree atrioventricular block.

3.2 Twenty-four hour Holter monitoring

Twenty-four hour Holter monitoring will be performed with time domain and frequency domain before HUTT at baseline and after 30 days treatment in all VVS patients.

Time domain parameters

1. standard deviation of NN intervals (SDNN)
2. standard deviation of all 5-min average NN intervals (SDANN)
3. square root of mean of the sum of squares of successive NN interval differences (rMSSD)
4. number of successive NN interval differing by >50ms divided by the total number of successive NN intervals (pNN50)

Frequency domain parameters

1. low frequency (LF): at frequency between 0.04--0.15 Hz
2. high frequency (HF): at frequency between 0.15--0.40 Hz
3. and the low frequency/high frequency ratio (LF/HF)

3.3 Detailed procedures of the modified Valsalva manoeuver (MV)

3.3.1 Expiration strength grades and breath-hold time of the modified MV.

Expiration strength is divided into 3 grades (Grade A = 20 mm Hg, Grade B = 30 mm Hg and Grade C = 40 mm Hg). Expiration strength is controlled by blowing into a tube connected to sphygmomanometer. Breath-hold time is divided into 2 lengths (1 = 8 seconds, 2 = 15 seconds).

3.3.2 Three steps of the modified MV

3.3.2.1 Threshold of MV

MVM group patients will be asked to perform MV in the order of A1, A2, B1, B2, C1 and C2 level. The VM level, which can be well tolerated without significant blood pressure and heart rate changes, is defined as threshold MV level for each individual patient.

3.3.2.2 Accommodation phase

The MV maneuvers will be performed in the morning session consisted 8 VM and in the afternoon session consisted 7 VM. Patients will be asked to perform VM from A1 level to C2 level gradually. Patients who do not reach the C2 level within 10 days will be excluded from the study.

3.3.2.3 Standardized MVM therapy phase

The MVM patients will be asked to perform 15 MV at C2 level daily about 50-60 minutes for 30 days.

3.4 Follow-up

Recurrence syncope of VVS patients will be observed during the follow-up of one year after 30 days therapy.

**Statistical analysis**

Continuous variables will be presented as mean ± standard deviation (SD). Continuous data with normal distribution will be assessed by Student’s *t*-test or one-way ANOVA with Post Hoc test (Bonferroni) as indicated, and non-normal distribution data will be tested by two-tailed Mann–Whitney U test or Kruskal-Wallis non-parametric test as indicated for the baseline characteristics of the three groups. The Chi-square test will be used to compare the ratio or percentages of categorical variables of the three groups. The Chi-square test will be used to compare the positive rate of HUTT at baseline and after 30 days treatment between NVM and MVM groups. The incidences of recurrent syncope during 12 months follow-up of NVM and MVM groups will be compared by log rank test of Kaplan-Meier curve. HRV indexes among the three groups will be assessed by one-way ANOVA with Post Hoc test (Bonferroni) for normal distribution data, Kruskal-Wallis non-parametric test for non-normal distribution data. HRV indexes between baseline and after 30 days treatment will be assessed by Student’s *t*-test for normal distribution data, two-tailed Mann–Whitney U test for non-normal distribution data. The relationship between HUTT and recurrent syncope, HRV and recurrent syncope during 12 months follow-up in VVS patients will be assessed by univariable and multivariable Cox proportional hazards models after 30 days intervention. Receiver operating characteristic (ROC) curves of HRV values at 30 days after intervention will be used to predict 12 months follow-up recurrent syncope. The cutoff values of HRV will be derived from ROC curve analysis by maximizing the sum of the sensitivity and specificity. All statistical analyses will be performed with SPSS version 19.0. *P*<0.05 is considered statistically significant.

**Expected results**

1 VVS患者存在自主神经功能失调紊乱。

The data will be similar at baseline among the three groups. Positive rate of HUTT will be lower in the MVM group than in the NVM group after 30 days treatment. The incidence of rate of recurrent syncope will be lower in the MVM group than in the NVM group during 12 months follow-up. VVS patients have autonomic nervous function imbalance by HRV analysis among the three groups and between baseline and after 30 days treatment. The underlying mechanism of MVM treating VVS is to improve autonomic nervous function.

**Study flowchart and Study plan**

**Study flowchart**

Syncope patient: HUTT, Holter and randomized

Healthy volunteers: Holter

MVM group: Exclude patients who cannot reach C2 within 10 days at accommodation phase. Conventional treatment plus standard MVM for 30 days

NVM group: conventional treatment for 30 days

CON group

HUTT, Holter after 30 days treatment. Incidence of recurrent syncope during 12 months follow-up

Holter after 30 days

**Study plan**

2012.05-2012.09 The clinical trial protocol, the ethical censor and clinical trial registration online.

2012.10-2014.12VVS patients’ recruitment, divide group, study process and 12 months follow-up.

2015.01- 2015.06 Study data statistical analysis and writing paper.

2015.07- Paper submission and publication.

**References**
