# Supplementary material for: A single-center randomized controlled trial observing the safety and efficacy of modified step-up graded Valsalva manoeuver in patients with vasovagal syncope
Source: PLoS One. 2018 Jan 30;13(1):e0191880. doi: 10.1371/journal.pone.0191880 (PMC5790265; doi:10.1371/journal.pone.0191880)
Supplement: S1 Ethics Committee Approval Document — (PDF) [file pone.0191880.s008.pdf]

武汉市普爱医院伦理委员会

临床试验审批件

【2012】伦审字（08-31-04）号

项目名称	改良 Valsalva 对血管迷走性晕厥治疗效果的临床观察					
临床试验机构	武汉市普爱医院/心内科			主要研究者	顾晔	
申办者	武汉市普爱医院/心内科					
审查类别	初始审查			审查方式	会议审查	
会议地点	武汉市普爱医院东院医技楼六楼会议室			会议时间	2012 年 8 月 31 日	
审查的文件	1 临床试验方案(版本号：1.0，版本日期：2012 年 8 月 20 号)<br>2 知情同意书(版本号：1.0，版本日期：2012 年 8 月 20 号)<br>3 病例报告表<br>4 研究者手册					
伦理审查意见	同意	作必要的修正后同意	作必要的修正后重审	不同意	终止或暂停已经批准的临床试验	回避
	7 票	0 票	0 票	0 票	0 票	0 票

经审查，该临床试验项目的科学性、伦理合理性符合要求，研究人员资质符合要求。同意按审查的研究方案进行临床试验。

年度/定期跟踪审查的频率从批准之日起每年一次。

武汉市普爱医院伦理委员会

主任委员（签名）：陈冠彦

日期：2012 年 9 月 5 日

声明

武汉市普爱医院伦理委员会的职责、人员组成、操作规程及记录遵循中华人民共和国食品药品监督管理局颁布的《药物临床试验质量管理规范》（GCP）的伦理审查原则，并遵守中国的有关法律及法规。

注意：

1 审查意见为“同意”的临床试验应遵循审查批准的试验方案执行，应符合SFDA/GCP和《赫尔辛基宣言》的原则。

2 审查意见为“作必要的修正后同意”的临床试验，应按审查意见对临床试验相关文件进行逐条说明、修改、补充，并在修改处明显标示，修改、说明、补充的文件连同初始审查意见一并提交伦理委员会快速审查。

3 审查意见为“作必要的修正后重审”的临床试验项目，应按审查意见对临床试验相关文件进行逐条说明、修改、补充，并在修改处明显标示，修改、说明、补充的文件连同初始审查意见一并提交伦理委员会会议审查。

4 审查意见为“不同意”和“暂停或终止”的临床试验，申办者和研究者可就伦理委员会的意见和建议中提及的问题进行书面申诉，并陈述理由。伦理委员会可就申诉作重新审查。

5 试验过程中对试验方案的任何修改均应提交伦理委员会审查批准后方可实施。

6 伦理委员会初始审查时根据试验的风险程度，决定年度/定期跟踪审查的频率。请在年度/定期跟踪审查到期前1个月提出年度/定期跟踪审查申请。

7 如果发生SAE，请在获知后24小时内报告SFDA和伦理委员会，并提交严重不良事件审查申请。

8 如果临床试验进行中发生不依从/违背方案的事件，请及时提交不依从/违背方案审查申请。

9 如果提前终止试验，请及时提交提前终止试验审查申请。

10 临床试验结束后，请及时提交结题审查申请。

爱医院 [illegible] 员会

地址：湖北省武汉市硚口区汉正街473号。邮编：430033。电话：027-68834993。
E-mail：pallwyh@126.com。

伦理委员会会议记录表

会议编号：紧急会议
会议日期：2012 年 8 月 31 日
会议时间：14:30 点开始
会议地点：武汉市普爱医院东院医技楼六楼会议室
会议主席：陈冠容
出席会议的伦理委员会委员名单

委员会职务	姓名	性别	工作单位	职务	签名
主任委员	陈冠容	男	武汉市普爱医院<br>临床药学研究所	所长	陈冠容
副主任委员	冯觉平	女	武汉市普爱医院<br>肿瘤科	主任	
副主任委员	王俊文	男	武汉市普爱医院<br>骨科	主任	王俊文
委员	曾繁典	男	华中科技大学同济医学院<br>临床药理研究所	教授	曾繁典
委员	周必光	男	武汉市普爱医院<br>骨科	主任	
委员	彭定凤	女	武汉市普爱医院<br>内科	主任	[illegible]
委员	黄利红	女	武汉市普爱医院<br>妇产科	主任	
委员	杨钟华	男	武汉市普爱医院<br>骨科	主任	
委员	田巧萍	女	武汉晚报	高级记者	田巧萍
委员	李丽平	女	湖北瑞通天元律师事务所	律师	李丽平
委员	李鹏	男	武汉市普爱医院<br>脊柱外科	主任	李鹏
委员	杜艳华	男	武汉市普爱医院<br>神经内科	主任	
委员	林梅	女	武汉市普爱医院<br>内分泌	主任	

…伦理委…
