# Supplementary material for: A single-center randomized controlled trial observing the safety and efficacy of modified step-up graded Valsalva manoeuver in patients with vasovagal syncope
Source: PLoS One. 2018 Jan 30;13(1):e0191880. doi: 10.1371/journal.pone.0191880 (PMC5790265; doi:10.1371/journal.pone.0191880)
Supplement: S2 Ethics Committee Approval Document — (DOCX) [file pone.0191880.s009.docx]

**Wuhan Puai Hospital Ethics Committee**

**Clinical trial approval document**

**[2012] Ethical review No (08-31-04)**

| Project name | The safety and efficacy of modified step-up graded Valsalva manoeuver in patients with vasovagal syncope | | | | | | | |
| --- | --- | --- | --- | --- | --- | --- | --- | --- |
| Clinical trial institution | Department of Cardiology, Wuhan Puai Hospital | | | | Principal investigator | | Ye Gu | |
| Application | Department of Cardiology, Wuhan Puai Hospital | | | | | | | |
| Investigate category | Initial Review | | | | Investigate mode | | Conference Review | |
| Conference site | 6th floor meeting room of Medical Technology Building, Wuhan Puai Hospital | | | | Conference date | | August 31, 2012 | |
| Review files | Clinical Trial Protocol (version number 1.0 ,version date August 20, 2012)  Patient Consent Form (version number 1.0 ,version date August 20, 2012)  Case Report Form  Investigator's Brochure | | | | | | | |
| Ethical review comments | Agree | Agree with the necessary amendments | Retrial with the necessary amendments | Disagree | | Termination or suspension of approved clinical trials | | Avoid |
|  | 7 | 0 | 0 | 0 | | 0 | | 0 |
| After investigation, the purpose of the clinical trial is scientific, ethical rationality meets the requirements, researcher qualification meets the requirements. Agree to conduct the clinical trial according to the review Clinical Trial Protocol.  The frequency of annual/regular track inspection is one year since the date of approval. | | | | | | | | |

Wuhan Puai Hospital Ethics Committee

Chairman (Signature)

Date

**The statement**

**The duties, personnel construction, operation procedures and records of Ethics Committee in Wuhan Puai Hospital follow the principles of ethical inspection in quality control standard for clinical trials (GCP) published by food and drug administration of the People's Republic of China , and abide by relevant laws and regulations of China.**

Note:

1. If review opinions of clinical trials are ‘Agree’, they should follow the review and approval of Clinical Trial Protocol, and comply with the principles of SFDA/GCP and the <Helsinki Declaration>.

2. If review opinions of clinical trials are ‘Agree with the necessary amendments’, they should explain, revise and supplement the relevant files according to review opinions one by one and marked on the modification. Then, submit together with the preliminary examination opinion to Ethics Committee for quickly inspection.

3. If review opinions of clinical trials are ‘Retrial with the necessary amendments’, they should explain, revise and supplement the relevant files according to review opinions one by one and mark on the modification. Then, submit together with the preliminary examination opinion to Ethics Committee for inspection.

4. If review opinions of clinical trials are ‘Disagree’ and ‘Suspension or termination’, the sponsors and researchers can appeal for problems mentioned by opinions and suggestions from the ethics committee and give their reasons in written form. Ethics Committee can inspect again for their application.

5. During the trial, any modification of trial protocol should be submitted to Ethics Committee. It can be practiced only after permitted.

6. At the initial review, Ethics Committee decides the frequency of annual/regular track inspection according to the level of risk, please apply for annual/regular track inspection one month before expiration.

7. If SAE happen, please report to the SFDA and the Ethics Committee within 24 hours after being informed, and submit an application for serious adverse events review.

8. If there is disobey/against events during clinical trials, please submit disobey/against plan in time.

9. If you want to stop the trial ahead of schedule, please submit an application for early termination trials review in time.

10. After finish the clinical trials, please submit an application for review in time.

Address: HanZheng Street 473^#^, QiaoKou District, Wuhan, Hubei Province.

Zip code: 430033 Telephone: 027-68834993 E-mail:pallwyh@126.com

**Ethics Committee Meeting Record Form**

Conference number: Emergency meeting

Conference date: August 31, 2012

Conference time: Start from 14:30

Conference site: 6th floor meeting room of Medical Technology Building, Wuhan Puai Hospital

Conference chairman: Chen Guanrong

List of ethics committee:

| Committee duties | Name | Gender | Work unit | Duty | Signature |
| --- | --- | --- | --- | --- | --- |
| Director | Chen Guanrong | Male | Clinical Pharmacology Research Department | Director |  |
| Vice director | Feng Jueping | Female | Oncology Department | Director |  |
| Vice director | Wang Junwen | Male | Orthopaedics Department | Director |  |
| Member | Zeng Fandian | Male | Clinical Pharmacology Research Department | Professor |  |
| Member | Zhou Biguang | Male | Orthopaedics Department | Director |  |
| Member | Peng Dingfeng | Female | Internal medicine Department | Director |  |
| Member | Huang Lihong | Female | Obstetrics and Gynecology Department | Director |  |
| Member | Yang Zhonghua | Male | Orthopaedics Department | Director |  |
| Member | Tian Qiaoping | Female | Wuhan Evening News | Senior Reporter |  |
| Member | Li Liping | Female | Hubei Ruitong Tianyuan Law Firm | Lawyer |  |
| Member | Li Peng | Male | Spine Surgery Department | Director |  |
| Member | Du Yanhua | Male | Neurology Department | Director |  |
| Member | Lin Mei | Female | Endocrine Department | Director |  |
